# A review of the online prognositc model predict using the POSH cohort (women aged ≤40 years at breast cancer diagnosis)

**DOI:** 10.1186/1745-6215-16-S2-P36

**Published:** 2015-11-16

**Authors:** Tom Maishman, Ellen Copson, Louise Stanton, Sue Gerty, Ed Dicks, Lorraine Durcan, Gordon Wishart, Paul Pharoah, Diana Eccles

**Affiliations:** 1 Cancer Sciences Academic Unit and University of Southampton Clinical Trials Unit, Southampton, UK; 2 Department of Oncology, University of Cambridge, Cambridge, UK; 3 Faculty of Medical Science, Anglia Ruskin University, Cambridge, UK

## Introduction

Breast cancer is the most common cancer women in the UK, with approximately 50,000 new cases each year. PREDICT (http://www.predict.nhs.uk) is an online prognostic tool developed to help determine the best available treatment and long-term outcome for early breast cancer. This study was conducted to establish how well PREDICT performs in estimating survival in a large cohort of younger women (aged ≤40 years) recruited to the POSH study.

## Methods

The UK POSH cohort includes data from 3000 women aged ≤40 years at breast cancer diagnosis. Study endpoints were overall- and breast cancer specific-survival at 5-,8-, and 10-years. Evaluation of PREDICT included model discrimination and comparison of the number of predicted versus observed events.

## Results

PREDICT provided accurate long term (8- and 10-year) survival estimates for younger women. However, short term (5-year) estimates were less accurate, with the tool overestimating survival by 25%, and by 56% for patients with ER positive tumours. PREDICT also underestimated survival at 5-years for patients with ER negative tumours.

## Conclusions

PREDICT is a user-friendly and reliable tool for providing accurate long-term survival estimates for younger women with breast cancer. However, the model requires further calibration for more accurate short-term estimates. Prediction in the short-term may be most relevant for the increasing number of women considering risk-reducing bilateral mastectomy.

